# Positive Facial Affect – An fMRI Study on the Involvement of Insula and Amygdala

**DOI:** 10.1371/journal.pone.0069886

**Published:** 2013-08-21

**Authors:** Anna Pohl, Silke Anders, Martin Schulte-Rüther, Klaus Mathiak, Tilo Kircher

**Affiliations:** 1 Department of Psychiatry, Psychotherapy, and Psychosomatics, Rheinisch-Westfälische Technische Hochschule Aachen University, Aachen, Germany; 2 Central Service Facility “Functional Imaging” at the Interdisciplinary Center for Clinical Research, Rheinisch-Westfälische Technische Hochschule Aachen University, Aachen, Germany; 3 Jülich Aachen Research Alliance – Translational Brain Medicine, Jülich/Aachen, Germany; 4 Department of Neurology, Rheinisch-Westfälische Technische Hochschule Aachen University, Aachen, Germany; 5 Department of Neurology, University Lübeck, Lübeck, Germany; 6 Cognitive Neuroscience, Institute of Neuroscience and Medicine, Research Center Jülich, Jülich, Germany; 7 Child Neuropsychology Section, Department of Child and Adolescent Psychiatry, Rheinisch-Westfälische Technische Hochschule Aachen University, Aachen, Germany; 8 Structural and Functional Organisation of the Brain, Institute of Neuroscience and Medicine, Research Center Jülich, Jülich, Germany; 9 Department of Psychiatry and Psychotherapy, Phillips-University Marburg, Marburg, Germany; University of California, San Francisco, United States of America

## Abstract

Imitation of facial expressions engages the putative human mirror neuron system as well as the insula and the amygdala as part of the limbic system. The specific function of the latter two regions during emotional actions is still under debate. The current study investigated brain responses during imitation of positive in comparison to non-emotional facial expressions. Differences in brain activation of the amygdala and insula were additionally examined during observation and execution of facial expressions. Participants imitated, executed and observed happy and non-emotional facial expressions, as well as neutral faces. During imitation, higher right hemispheric activation emerged in the happy compared to the non-emotional condition in the right anterior insula and the right amygdala, in addition to the pre-supplementary motor area, middle temporal gyrus and the inferior frontal gyrus. Region-of-interest analyses revealed that the right insula was more strongly recruited by (i) imitation and execution than by observation of facial expressions, that (ii) the insula was significantly stronger activated by happy than by non-emotional facial expressions during observation and imitation and that (iii) the activation differences in the right amygdala between happy and non-emotional facial expressions were increased during imitation and execution, in comparison to sole observation. We suggest that the insula and the amygdala contribute specifically to the happy emotional connotation of the facial expressions depending on the task. The pattern of the insula activity might reflect increased bodily awareness during active execution compared to passive observation and during visual processing of the happy compared to non-emotional facial expressions. The activation specific for the happy facial expression of the amygdala during motor tasks, but not in the observation condition, might reflect increased autonomic activity or feedback from facial muscles to the amygdala.

## Introduction

Imitation is an ability that includes a wide range of different phenomena. Infants imitate nearly from birth on [1] and learn through imitation during development [2]. In social situations, humans perceive the interaction partner as more likable, and the interaction as more successful, if their non-verbal behaviour is imitated by their partner [3]. Being imitated in contrast to not being imitated increases neural activity in brain regions associated with reward [4]. Thus, the automatic tendency to mimic an interaction partner's body posture, tone of voice, and facial expression is likely to increase the success of social interactions and functions as ‘social glue’ [5]. Emotional cues are of high importance for imitation. It was shown that participants mimic emotional facial expressions automatically even when they are instructed to observe emotional faces without moving [6], or when the emotional faces were presented subliminally [7]. Performing incongruent facial expressions suppresses this automatic tendency and slows the reaction times for the execution of the facial expressions [8].

In previous studies, to examine the neural basis of imitation, participants were explicitly asked to reproduce facial expressions (e.g. [9], [10]). Explicit imitation of movements was shown to involve several brain regions beyond motor and sensorimotor cortex. Visual input is claimed to be forwarded via the superior temporal sulcus to the rostral inferior parietal lobule (IPL) and then to the pars opercularis of the inferior frontal gyrus (IFG op) [9], [11]. The latter two regions are believed to form the human ‘mirror neuron system’ and to play a key role during the transfer of perceptual information into motor output [12]. During observation of facial expressions IPL and IFG op are believed to start to ‘resonate’ which means that an internal motor representation of the expression is generated [13], [14]. The ‘resonance’ may serve different functions such as action understanding [14] and facilitation of motor output during imitation [15].

Beyond the putative human mirror neuron system and motor-, as well as sensorimotor cortices, the pre-supplementary motor area (pre-SMA) was reported to be involved in imitation of emotional facial expressions [9], [13], [16]. Furthermore, the insula and the amygdala were hypothesized to be part of an emotional perception-action matching system [11], [17] and therefore to “extend” the classical MNS (which was shown to respond to goal directed hand movements) during emotion processing [13]. Both regions were previously reported to be involved in observation and imitation of emotional facial expressions (angry, happy, fearful, sad, disgusted, surprised [9]). Interestingly, right insula activation was predicted by the magnitude of facial movements of participants (angry, sad and happy; when exclusively masked with non-emotional facial expressions). Moreover, left amygdala activation was predicted by extent of movement during imitation of happy facial expressions [10]. In contrast, no activation of insula and amygdala was reported during observation (angry, happy, fearful, disgusted, neutral [18]) and imitation of emotional facial expressions in two further fMRI studies (angry and happy [16]). Van der Gaag and colleagues [13] found increased bilateral anterior insula activation but did not find increased amygdala activation during observation of emotional facial expressions (happy, disgusted and fearful, all emotions pooled together) when contrasted with observation of non-emotional facial expressions (blowing cheeks). In a further analysis of their data the authors compared the BOLD signal of the amygdala during observation between disgusted, fearful, happy and non-emotional (blowing cheeks) facial expressions but found no significant differences. This was true for passive observation, observation for discrimination of facial expressions and observation for delayed imitation (motor aspects were not included in the general linear model). Significant differences of amygdala activation were noted only when observation of facial expressions was compared to observation of patterns but amygdala activation was not specific to emotional processing [19]. Hennenlotter and colleagues [14] showed involvement of the left anterior insula during both observation and execution of pleasant facial affect. The bilateral amygdala was also involved in both tasks, but activation sites differed. During observation the bilateral ventral amygdala was activated, whereas the bilateral dorsal amygdala was involved in smile execution. Contrary, shared representations of observation and execution of happy facial expressions were found in the same part of the bilateral amygdala [20].

In sum, both regions seem to be involved during imitation, perception and execution of emotional facial expressions, but results of studies differ, which may be due to differences in the task (observation with different tasks, execution, (delayed) imitation), stimulus material (pictures versus video clips of different length), control condition (fixation cross, neutral face without movement, non-emotional facial expression) or analysis (e.g. conjunction of two t maps versus separate listing of two t-tests, different thresholds).

We aimed at examining brain regions with increased BOLD response during imitation of positive facial affect. We wanted to control for effects due to motion and therefore compared the happy facial expression with a non-emotional facial expression. We hypothesized to find amygdala and insula involved in affective imitation. In subsequent analyses we intended to compare the BOLD responses of the target regions also during perception and execution of facial expressions, respectively. We focused on the insula and the amygdala, because these structures were assumed to be central for the “extended” MNS [13] and respond consistently to emotional stimuli (for a review see [21]).

## Methods

### Ethics Statement

The study was approved by the local Ethics Committee (Medical Faculty of the RWTH Aachen University; code: EK 099/08) based on the declaration of Helsinki and all participants gave written informed consent prior to participation. They were paid for their participation.

### Participants

Thirty-two healthy, right-handed [22] volunteers (17 women) with no history of neurological or psychiatric disease participated in the study. Participants had normal or corrected-to-normal vision. Five participants had to be excluded from the analysis because of intolerable head-movements or inaccurate completion of the task (see also Design/Task and fMRI Acquisition Parameters and Data Analyses). Accordingly, 27 out of 32 participants were included in the final analyses (mean age: 24.6 years old (*SD* = 5.4); averaged school education: 12.9 years (*SD* = 0.6)). Data from all subjects were published in a previous study focusing on shared representations during observation and execution of facial expressions [20].

### Stimuli

**Figure 1:**
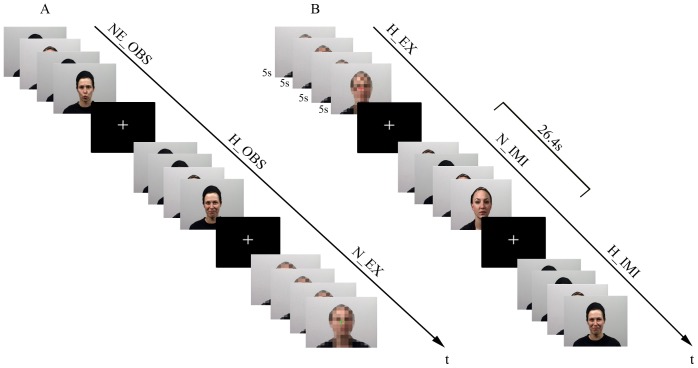
Video clips were presented in all conditions. In the first and second run (A) participants observed and executed facial expressions (if an actor or scrambled faces were displayed, respectively), in the third and fourth run (B) participants were to imitate facial expressions if an actor was presented, and again, to execute facial expressions in case of scrambled video clips. Within one block, four video clips à five seconds were shown. Abbreviations: A) N_OBS: non-emotional observation, E_OBS: emotional observation, C_EXP: control execution, B) E_EXP: emotional execution, C_IMI: control imitation, E_IMI: emotional imitation.

We used video clips depicting facial expressions as dynamic stimulus material. The clips were recorded in an in-house media centre with a commercial video camera (Sony DVX 2000, spatial resolution 720×576 pixels). The video clips depicted 24 actors executing happy facial expressions (smile), non-emotional facial expressions (lip protrusion), or neutral faces (relaxed face without motion). Each video clip began with the actor having a neutral face. After one second, the actor began to produce a facial expression. The video clip ended again with a neutral face (see Figure 1; the actors have given informed consent to publish the case details.). The total length of each video clip was 5 seconds. We also created pixelated versions of each video, where moving squares created a ‘scrambled’ version of the faces (down sampled pictures had 1/40 of the original resolution) so that only the silhouette of the actor was recognizable (Photoshop CS3 v.10.0 ® and Adobe Premiere Pro CS3 ®) (Figure 1). After one second, a colored fixation cross (red, blue, or green) was projected onto the scrambled video for 3 seconds to cue the participant for execution of the facial expression (see below). Altogether, 72 video clips showing facial expressions and 24 video clips with scrambled facial silhouettes were used in the present study. The stimuli were presented with MR-compatible goggles (Resonance Technology, Inc. Northridge, CA) using the Presentation© software package v.11.0 (Neurobehavioral Systems, Inc., Albany, CA). The evaluation of the stimulus material is described in detail Kircher and colleagues [20].

### Design/Task

Before the fMRI experiment participants were told that they would see either video clips depicting actors showing a facial expression (smile, lip protrusion, neutral face), or scrambled faces. For run 1 and 2, they were instructed to (i) attentively *observe* video clips depicting actors, and to (ii) *execute* a facial expression (for the duration of the colored fixation cross) when scrambled faces were presented. The color of the fixation cross indicated which facial expression participants were to execute (smile, lip protrusion, neutral face). In advance to the third and forth run, participants were instructed to immediately (iii) *imitate* the facial expressions in the video clips (instead of solely observing it). They were also told that the task in case of the scrambled videos remained the same as before (*execute*) (Figure 1).

Accordingly, the imitation task was presented separately from the observation task in the last two runs of the experiment and participants did not know about the imitation condition until run 3. This order was chosen to prevent participants from unintentionally imitating already during the observation task.

The fMRI experiment was designed as a two factorial design with the factors task (*Observation, Execution, Imitation*) and facial expression (*Happy Facial Expression, Non-Emotional Facial Expression, Neutral Face*). Videos of neutral faces without motion served as high-level baseline for happy and non-emotional facial expressions. We included only one emotional facial expression, because of time restrictions of fMRI measurements. We chose to examine happy facial expressions representative for emotional facial expressions, because the happy facial expression may be the easiest to identify and perform. As the variability to execute angry and sad facial expressions is rather high, the movement congruency between observation and execution is highest for happy facial expressions (see also [14]). Thus, differences in brain activation between tasks would not be due to effects of incongruence of movements.

The videos were presented in blocks of four 5 s-video clips. Each run consisted of 18 blocks of video clips (3 blocks of each condition). The task order within the single runs was pseudo-randomized across participants. A low-level baseline (fixation cross) was presented between the blocks for 6.4 seconds (one block plus low level baseline corresponded to 12 scans). After every sixth block an additional baseline (30.8 seconds corresponding to 14 scans) was included to allow for the hemodynamic response to return to baseline. All together, the fMRI scanning lasted for approximately 45 min.

The faces of the participants were video-taped during the entire fMRI experiment to ensure that participants followed the task instructions. The video tapes were judged online and after the experiment by a certified Facial Action Coding System rater (FACS; [23]). The experiment was interrupted if a participant mimicked the actor's facial expression during the observation task or executed the wrong facial expression during the execution task. One participant had to be excluded from further analysis because of repeated imitation reactions during the observation task.

After fMRI scanning, participants completed a post-scanning rating of their subjective feeling of happiness during all conditions. The rating was presented on a 7-point Likert scale (1 = ‘not at all’ until 7 = ‘very strong’). A paired t-test was calculated to test if participants felt happier in the emotional than in the non-emotional imitation condition.

### fMRI Acquisition Parameters and Data Analyses

Functional T2* weighted images were obtained with a Siemens 3-Tesla MR-scanner using Echo planar imaging (EPI) (TR = 2200 ms, TE = 30 ms, flip angle 90°, FoV 224 mm, base resolution 64, voxel size 3.3×3.3 mm^2^, 36 slices with slice thickness 3.5 mm, and distance factor 10%).

High-resolution T1-weighted anatomical 3-D Magnetization Prepared Rapid Gradient Echo (MP-RAGE) images (TR 1900 ms, TE 2.52 ms, TI 900 ms, flip angle 9°, FoV 250 mm, 256×256 matrix, 176 slices per slab) were acquired at the end of the experimental runs.

Data processing and statistic analyses (see also [20]) of the imaging data were performed using SPM5 (Wellcome Department of Imaging Neuroscience, London, UK) implemented in Matlab 7.2 (Mathworks Inc., Sherborn, MA, USA). The first five EPI volumes were discarded to allow for T1 equilibration. The remaining functional images were realigned to the first image to correct for head motion [24]. Head motion of less than 4 mm and less than 3° was accepted. Four subjects did not meet these requirements and were therefore excluded from further analyses (see above). For each participant, the T1 image was co-registered to the mean image of the realigned functional images. The mean functional image was normalized to the MNI template (Montreal Neurological Institute, [25], [26] using a segmentation algorithm [27]. All images were normalized, resampled to 1.5×1.5×1.5 mm^3^ voxel size and spatially smoothed with an 8-mm full-width half-maximum (FWHM) isotropic Gaussian kernel.

Data were subsequently analyzed by a two-level approach. Using a general linear model (GLM), each experimental condition was modeled on the single-subject level with a separate regressor convolved with a canonical hemodynamic response function and its first temporal derivative. The following conditions were included:

1. *Happy_Observation (H_OBS)*
2. *Non-Emotional_Observation (NE_OBS)*
3. *Neutral_Observation (N_OBS)*
4. *Happy_Execution (H_EX)*
5. *Non-Emotional_Execution (NE_EX)*
6. *Neutral_Execution (N_EX)*
7. *Happy_Imitation (H_IMI)*
8. *Non-Emotional_Imitation (NE_IMI)*
9. *Neutral_Imitation (N_IMI)*

The low-level baseline between the experimental conditions (fixation cross) was implicitly modeled as baseline. Realignment parameters were included as six additional regressors in the GLM as head motion nuisance covariates. The execution task was present in all four runs and therefore twice as often as the other tasks. However, to avoid effects due to habituation and repetition, only the execution blocks of the first and second run were included in the second level analysis resulting in an equal amount of blocks of each experimental condition.

Parameter estimates for each voxel were calculated using maximum likelihood estimation and corrected for non-sphericity. First-level contrasts were fed into a flexible factorial second level group analysis with the factors condition and subjects. The factor condition was modeled as fixed effect and encompassed all 9 levels of the single-subject analysis. The subjects-factor was modeled as random effect.

The first six conditions of the experiment are described detailed in Kircher et al. [20]. In this previous study, conjoint activations of observation and execution were presented. Shared representations for the happy facial expressions were contrasted with shared representations for the non-emotional facial expressions to examine the specificity of shared circuits of positive affect.

First, to show the reliability of our experimental design, we examined activations during imitation of happy and non-emotional facial expressions, separately. Accordingly, two contrasts were computed: imitation of happy facial expressions was contrasted with the high level baseline (H_IMI>N_IMI), likewise imitation of the non-emotional facial expressions was contrasted with the high level baseline (NE_IMI>N_IMI). We hypothesized to replicate activation patterns of previous studies examining the imitation of facial expressions.

Next, we tested for activations specific for the imitation of happy facial expressions, namely, activation during imitation of the happy facial expressions contrasted with the activation during imitation of the non-emotional facial expressions (H_IMI>NE_IMI). All whole brain analyses at group level are reported as significant at a threshold of p<.05, FWE corrected (k>15). Brain structures were labeled using the Anatomy Toolbox v 1.6 [28], [29] and the WFU PickAtlas software (Wake Forest University, Winston-Salem, NC; [30]). Moreover, we were interested in examining the involvement of the insula and the amygdala in facial expression observation and execution. Therefore, we extracted data at the peak voxel of the contrast H_IMI>NE_IMI for six conditions of interest. The conditions encompassing neutral facial expressions served as high-level baseline for the reliability analysis and analyses presented in Kircher et al. [20] and were not of interest for the continuative analyses, because participants did not move during execution and imitation. A repeated-measurement two-way ANOVA (within-subject factors task (observation, execution, imitation) and expression (happy, non-emotional) was calculated for each region using SPSS 15.0 (Statistical Packages for the Social Sciences, SPSS Inc., USA).

## Results

### Rating

To identify differences in emotional experience during the imitation tasks, subjective ratings of happiness were compared using a paired t-test. Participants reported a stronger feeling of happiness during imitation of emotional facial expressions (*M* = 5.27, *SD* = 1.31) than during imitation of non-emotional facial expressions (*M* = 2.88, *SD* = 1.42); *t(26)* = 6.48, *p*<0.001). The same was true for the observation end execution conditions. Results of these conditions were already presented in Kircher and colleagues [20].

### FMRI Data: Whole Brain Analyses

**Figure 2:**
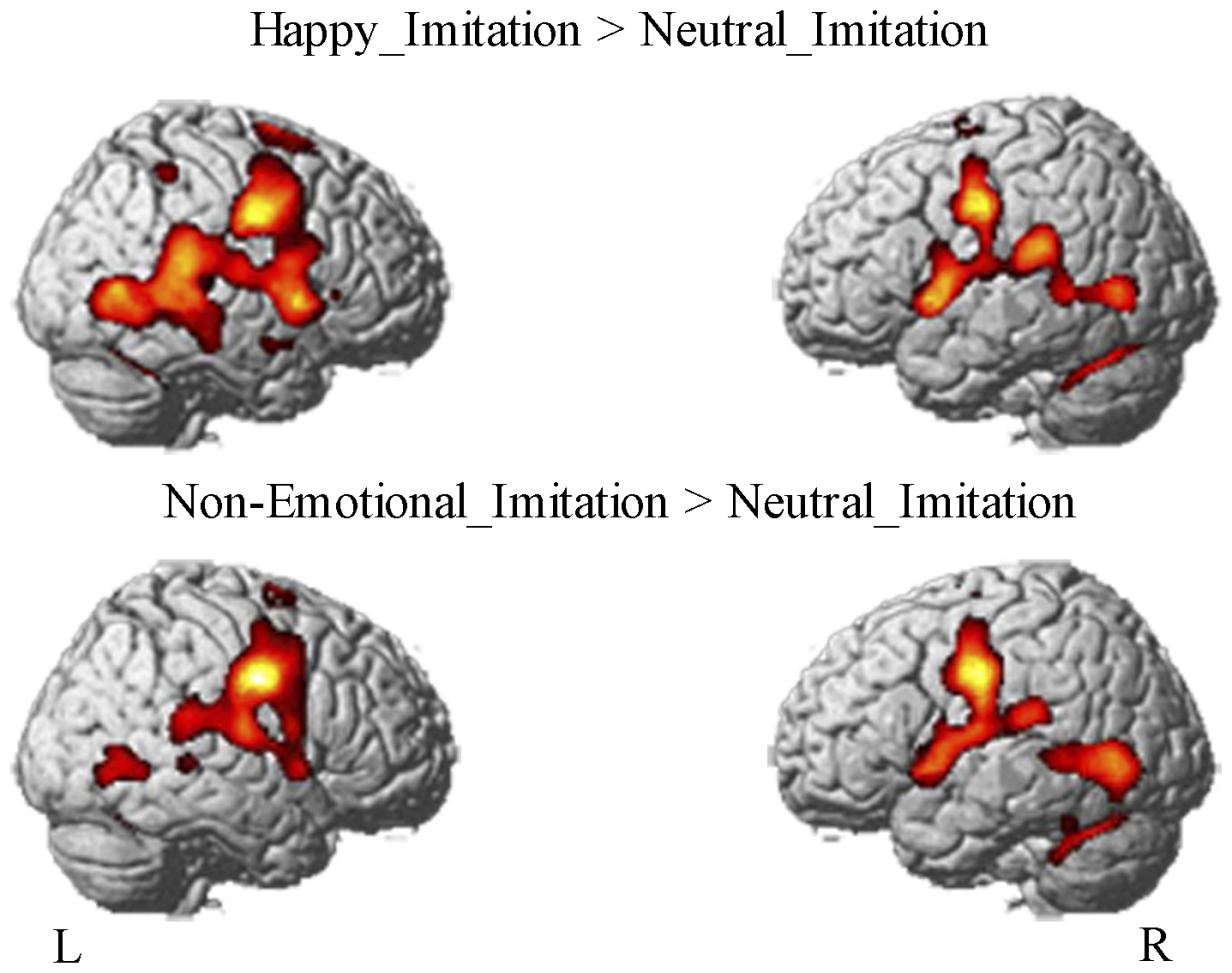
Neural network underlying imitation of emotional and non-emotional facial expressions. The analysis was FWE-corrected at a threshold of p<0.05 (k>15). Both facial expressions involved a widespread bilateral network including (pre-)motor areas, the insula, temporal areas, the brain stem, and the cerebellum.

Imitation of both facial expressions revealed a distributed network mainly including frontal and parietal cortices (see Table 1 and Figure 2). Activations were found in the bilateral temporal, (pre-) motor and somatosensory cortex. In particular, the post- and precentral gyrus (BA 3, 4, 6) and the pre-supplementary motor area (pre-SMA, BA 6) extending to medial cingulate cortex were involved. Furthermore, imitation was associated with bilateral activation in the posterior middle temporal gyrus (MTG), the cerebellum, and the bilateral insula. For emotional facial expressions, insula activation extended into the right pars opercularis of the inferior frontal gyrus (IFG op, BA 44), as well as the into right pars triangularis (BA 45). Additionally, the posterior parietal cortex including area PFm of the inferior parietal lobule (IPL), 7PC, and hiP2 of the superior parietal lobule (SPL) (for localization of the cytoarchitectonic regions PFm, 7PC, and hiP2 see [31]) was activated.

**Table 1:** Activation clusters for imitation of emotional and non-emotional facial expressions.

	MNI							
Contrast	X	Y	Z	k	t-value	p-value	Side	Region
Imitation: Happy>Neutral	54	−8	36	23044	12.48	<0.001	R	Postcentral gyrus
	−46	−12	36	11240	12.55	<0.001	L	Postcentral gyrus;
	3	2	57	5582	13.58	<0.001	B	pre-SMA
	−24	−62	−22	3821	10.94	<0.001	B	Cerebellum (lobule IV, VIIa)
	−3	−30	−9	150	6.06	<0.001	L	Brain stem
	48	−44	54	143	5.92	0.001	R	IPL (PFm)
	50	−4	−16	90	5.51	0.003	R	MTG
	−8	−12	−21	27	5.21	0.013	L	Brainstem
	44	26	3	24	5.03	0.015	R	IFG pars triangularis (BA 45)
Imitation: Non-Emotional>Neutral	57	−6	36	9787	16.95	<0.001	R	Postcentral gyrus (BA 3)
	−54	−9	38	9008	16.30	<0.001	L	Postcentral gyrus (BA 3)
	2	−2	57	3460	10.66	<0.001	R	SMA
	−50	−68	0	2983	9.31	<0.001	L	MTG
	−22	−62	−22	2098	11.78	<0.001	L	Cerebellum VI
	22	−63	−22	959	9.72	<0.001	R	Cerebellum VI
	46	−58	0	937	7.05	<0.001	R	Posterior MTG
	−14	−12	−3	474	9.29	<0.001	L	Thalamus
	52	−38	0	104	5.92	0.001	R	MTG
	9	12	34	35	5.35	0.007	R	MCC

*The table shows MNI coordinates of the main peaks of significant clusters, the number of significant voxel (k), FWE-corrected, p-values<0.05, k>15, the hemisphere (L = left, R = right, and B = bilateral), and the name of the region in which the main peak was localized. Abbreviations: pre-SMA: pre-supplementary motor area, IPL: inferior parietal lobule, MTG: middle temporal gyrus, IFG: inferior frontal gyrus, MCC: middle cingulate cortex.*

**Figure 3:**
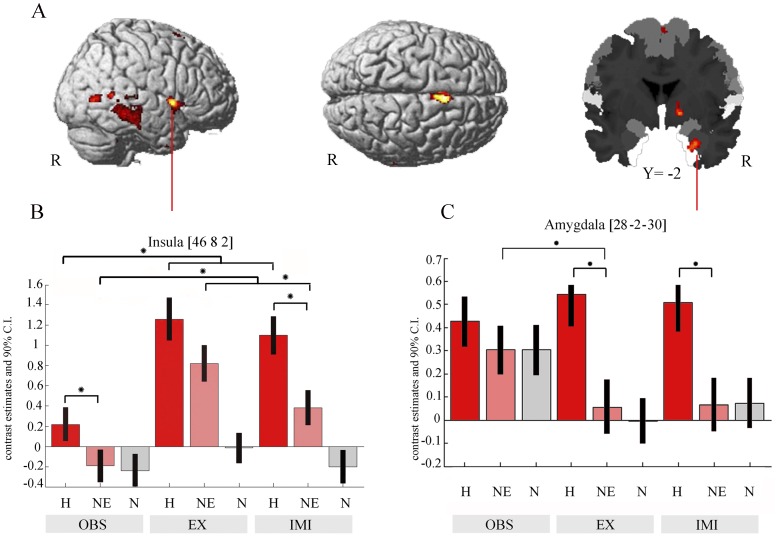
Affect-specific activation during imitation as revealed by direct comparison of the emotional and the non-emotional facial expression (FWE-corrected at a threshold of p<0.05; k>15). Significant right-hemispheric activation differences were found, amongst others, right-hemispheric in the pre-SMA, the insula, the amygdala, and the MTG (A). Average parameter estimates extracted from the main peak and depicted including 90% confidence interval (CI). The emotional conditions (E) are colored in red, the non-emotional (N) in rose, and the control (neutral face without movement (C)) in grey. Significant post-hoc t-tests (p<0.0055 with Bonferroni correction) are marked with an asterisk (B).

Imitation of happy in contrast to non-emotional facial expressions (H_IMI>NE_IMI) was associated with right hemispheric activation in the pre-SMA, the right insula extending to the IFG op (BA 44) and the pars triangularis (BA 45), and in the right amygdala extending to the parahippocampal gyrus. Furthermore, increased activation was found in the right middle temporal gyrus (MTG) extending to the inferior temporal gyrus and to the visual area V5, in the right thalamus, in the right pallidum and in the right cuneus (see Table 2 and Figure 3)

**Table 2:** Activation clusters specific for the emotional facial expressions (Contrast: Happy_Imitation>Non-Emotional_Imitation).

MNI							
X	Y	Z	k	t-value	p-value	Side	Region
50	−30	−15	1206	7.54	<0.001	R	MTG
3	10	57	625	7.26	<0.001	R	pre-SMA
39	−45	6	371	6.27	<0.001	R	MTG
46	8	2	366	6.42	<0.001	R	Insula
24	−26	3	301	6.48	<0.001	R	Thalamus
29	−2	−30	149	6.06	<0.001	R	Amygdala
15	−2	−3	104	6.26	<0.001	R	Pallidum
54	−51	8	75	5.30	0.009	R	MTG
18	−75	20	65	5.30	0.009	R	Cuneus

The table shows MNI coordinates of the main peaks of the significant clusters, the number of significant voxel (k), FWE-corrected p-values<0.05, k>15, the hemisphere (L = left, R = right, and B = bilateral), and the name of the region in which the main peak was localized. Abbreviations: MTG: middle temporal gyrus, pre-SMA: pre-supplementary motor area.

### FMRI Data: Analyses of Insula and Amygdala Activation

To compare the activation of the right anterior insula in six conditions of interest, contrast estimates were extracted at the peak voxel in whole brain analysis H-IMI>NE_IMI (MNI [46 8 2]) from the normalized and 8 mm smoothed images and entered into a repeated measures ANOVA with factors task (observation, execution, imitation) and facial expression (happy, non-emotional). This analysis revealed a significant main effect for the factor facial expression, *F*(1, 26) = 38.6, *MSE* = 0.45, *p*<0.001, as well as for the factor task, *F*(2, 52) = 30.96, *MSE* = 0.70, *p*<0.001. There was no significant interaction between the two factors, *F*(2, 52) = 0.64, *MSE* = 0.33, *p* = 0.53. The Bonferroni-corrected threshold of dependent post-hoc *t*-tests was *p*<0.0055. Post-hoc tests between conditions revealed significant stronger activation when facial expressions were imitated or executed than when they were only observed. Furthermore, execution of the non-emotional facial expressions revealed stronger insula activation compared with imitation of the non-emotional facial expression. Observation of the happy facial expressions resulted in significantly stronger insula activation than observation of the non-emotional facial expressions. The same was true for imitation, which was already shown in the whole brain analysis (for means, standard deviations and post-hoc tests see Table 3).

**Table 3:** Analyses of insula and amygdala activation.

	*Insula*		*Amygdala*	
Condition	*M*	*SD*	*M*	*SD*
Happy_Observation	0.17	0.73	0.45	0.3
Happy_Execution	1.45	0.96	0.52	0.64
Happy_Imitation	1.18	0.53	0.57	0.53
Non-Emotional_Observation	−0.37	0.68	0.32	0.39
Non-Emotional_Execution	0.82	0.80	−0.01	0.51
Non-Emotional_Imitation	0.39	0.72	0.09	0.42

Post Hoc Tests Insula	*t*	*df*	*p*
Happy: Imitation>Observation	−5.85	26	<0.001
Non-Emotional: Imitation>Observation	−3.449	26	0.002
Happy: Execution>Observation	−5.05	26	<0.001
Non-Emotional: Execution>Observation	−5.487	26	<0.001
Non-Emotional: Execution>Imitation	3.208	26	0.004
Observation: Happy>Non-Emotional	4.135	26	<0.001
Imitation: Happy>Non-Emotional	5.625	26	<0.001

Post Hoc Test Amygdala	*t*	*df*	*p*
Imitation: Happy>Non-Emotional	5.167	26	<0.001
Execution: Happy>Non-Emotional	4.821	26	<0.001
Non-Emotional: Observation>Execution	3.100	26	0.005

Top: The table shows means (M) and standard deviations (SD) of extracted data from main peak activation of insula (MNI [46 8 2]) and amygdala (MNI [28 −2 −30]) in the comparison Emotional_Imitation>Non-Emotional_Imitation). Bottom: The table shows t-values degrees of freedom (df) and p-values of post-hoc t-tests for both regions of interest (p<0.0055 with Bonferroni correction).

For the amygdala, contrast estimates were extracted from the same contrast (H_IMI>N_IMI) at the peak voxel in the whole brain analysis (MNI [28 −2 −30]) from the normalized and 8 mm smoothed images and entered into a repeated measures ANOVA with factors task (observation, execution, imitation) and facial expression (emotional, non-emotional). The repeated measures ANOVA revealed a significant main effect for the factor facial expression, *F*(1, 26) = 41.053, *MSE* = 0.14, *p*<0.001 but not for the factor task, *F*(2, 52) = 0.93, *MSE* = 0.23, *p* = 0.401. There was a significant interaction between facial expression and task, *F*(2, 52) = 6.77, *MSE* = 0.1, *p* = 0.002. The post hoc *t*-tests (Bonferroni corrected *p*<0.0055) indicated that the right amygdala showed significantly stronger activation during observation of the non-emotional facial expressions than during execution of the non-emotional facial expressions. Execution of happy facial expressions led to significantly stronger right amygdala activation than execution of the non-emotional facial expressions. The same was true for the imitation condition, which was already shown by the whole brain analysis but not for the observation condition, *t*(26) = 1.89, *p* = 0.07.

No other comparison survived the Bonferroni correction (but there were two trends: Insula activation tended to be stronger during execution of the happy, compared with execution of the non-emotional facial expressions, *t*(26) = 2.96, *p* = 0.006. Amygdala activation tended to be stronger during observation of the non-emotional facial expressions in comparison to imitation of the non-emotional facial expressions, *t*(26) = 2.48, *p* = 0.020) (see also Table 3).

In summary, analyses of the fMRI data revealed the following results (see Figure 3): First, imitation and execution of facial expressions involved the right insula significantly stronger than observation of facial expressions did. Secondly, the right insula showed significant stronger activity for the happy than for the non-emotional facial expressions, irrespective if facial expressions were observed or imitated. Third, the right amygdala showed an increased difference between the happy and the non-emotional facial expressions during imitation and execution in comparison to observation.

## Discussion

We examined neural correlates specific for happy in comparison to non-emotional facial expressions during imitation. In line with previous research, happy and non-emotional facial expressions activated a similar bilateral imitation network encompassing (pre-)motor, somatosensory, and superior and middle temporal cortices. Imitation of happy facial expressions contrasted with imitation of non-emotional facial expressions revealed right hemispheric activity in the pre-SMA, insula extending to premotor cortices, the amygdala and the medial temporal cortex.

### Insula and IFG op

Involvement of the insula in emotional tasks has been shown in several studies (for reviews see [21], [32]). Anders and colleagues [33] for example showed that increased activation of the right insula is correlated with an increase in the experience of negative valence. There is growing consensus that the insular cortex mediates the experience of emotions by representation of changes of internal physical states [21]. Critchley and colleagues [34] showed insula involvement during the explicit access to visceral information. Activation of the right insula correlated positively with the accuracy to detect one's own heart beats and efficiency in the heart beat task was associated with trait ratings of negative affect. The involvement of the right anterior insula during subjective ratings of changes in bodily states (here temperature changes) was also shown by Craig and colleagues [35].

We found significantly stronger insula activation during both imitation and observation of happy in comparison to the non-emotional facial expressions (with a trend concerning the execution of facial expressions). The awareness of bodily states might be stronger, when an emotionally salient stimulus is presented (here the actor depicting a happy facial expression). Summing up our results, insula activation was significantly stronger during motor tasks and when an emotionally salient stimulus was presented. Both effects are in line with Craig [32] claiming a specific role of this region during awareness in general.

In a previous Magnetencephalography study Chen and colleagues [36] asked participants to put themselves in the emotion of visually presented facial expressions. An increase of insula activation was found for happy and disgusted facial expression exposure in comparison to neutral faces. Significant stronger activation of right anterior insula during disgust in comparison to happiness was also shown but relatively late after stimulus onset. The authors speculated that insula activation may decline earlier during visually induced pleasant emotions than during observation of emotional facial expressions with negative valence. A faster decline of insula response during observation in comparison to execution/imitation of facial expressions might also be due to differences of internal states between tasks. This maybe explains the significant differences between perceptive and active tasks in our study. Further MEG studies with high temporal resolution would be needed to examine differences in time response of the insula in the different tasks.

Interestingly, activation of the insula specific for happy affect extended to the IFG op (BA 44) and pars triangularis (BA45). Several studies have found activation in IFG op during imitation of hand-movements (e.g. [37]) or during imitation of emotional facial expressions [9], [13], [16]. IFG op is part of the putative human mirror neuron system, which is believed to mediate action understanding [12], [14] by decoding the goal of an observed action [38], [39]. Evidence for the importance of the IFG op for imitation came from a transcranial magnetic stimulation (TMS) study, which found that perturbation of left and right IFG op significantly increased error rates in a finger imitation task [15]. But it is still under debate if this area plays a pivotal role in imitation (e.g. [9], [37], [40], [41]), or if its involvement in imitation is overestimated [42]. For example, IFG op activity might be confounded by other cognitive functions like execution timing [43]. Importantly, in our study execution timing was needed during imitation of both facial expressions. Therefore, timing issues cannot explain the increased activation for the happy (as compared to the non-emotional) facial expressions. In a recent study, a cluster encompassing the right anterior insula and the adjacent frontal operculum was triggered by IFG (BA 45) activation during observation and experience of disgust [44]. Combining these results and the model of neural correlates of imitation where visual input is forwarded through STS, IPL and then IFG op [9], [11], we hypothesize that IFG might trigger increased insula activation during affective imitation.

### Amygdala

The amygdala, a central part of the emotion-circuitry (for a review see [45]), was similarly activated during observation of emotional and non-emotional facial expressions. Thus, the finding of van der Gaag et al. [19] was replicated by our results. A meta-analysis of Sergerie and colleagues [45] revealed general stronger effect sizes of amygdala activation during observation of faces compared with pictures. The amygdala might act as a ‘relevance detector’ during observation of biologically relevant stimuli [46].

While the difference between the emotional and non-emotional facial expressions was small during observation, we found affect-specific increase of the right amygdala during imitation and execution. Executing emotional facial expressions has been claimed to increase autonomic arousal and emotional experience [47]. Phillips and colleagues [48] argued that the autonomic response during the experience of an affective state might be mediated by amygdala activation. In line with this, infusing a γ-aminobutyric acid (GABA-A) antagonist into the amygdala of rats led to increased blood pressure and heart rate [46]. Moreover, the amygdala is reciprocally connected to the hypothalamus and brain stem regions involved in autonomic control [49]. In the current study, the autonomic response might have been stronger during the execution of emotional facial expressions than during the execution of non-emotional facial expressions.

In another line of evidence, a recent study by Hennenlotter and colleagues [50] showed that facial feedback during imitation of emotional facial expressions modulates amygdala activation. When feedback of facial muscles was inhibited by treatment with botulinum toxin, left amygdala activation decreased. Additionally, contraction intensity of facial muscles correlated with left amygdala activity when muscles were not treated with botulinum toxin. Also Lee and colleagues [10] found correlations between the magnitude of facial movements during imitation of happy facial expressions and activation of the left amygdala. Thus, increased affect-specific amygdala activity during the motor tasks in the current study could also be a result of increased facial feedback during emotional motor conditions.

Finally, it should also be mentioned that affect-specific activation of amygdala during the emotional motor conditions might also be due to faster habituation of the amygdala during observation of facial expressions. While amygdala response has been shown to habituate during observation of emotional facial expressions (e.g. [51]), experiencing emotions did not lead to amygdala habituation [52]. Thus, affect-specific amygdala activation during the emotional motor tasks in comparison with observation may be caused by habituation of amygdala during observation, especially in blocked stimulus presentation designs.

### Lateralization

We found bilateral activations during imitation of positive and non-emotional facial expressions. Although possible lateralization during imitation has been debated controversial [53], a bilateral network is in line with a recent meta-analysis on imitation. Notwithstanding, it has to be noted that most studies included in this meta-analysis examined hand movements (29 out of 35) and only few explored facial expressions [53].

When imitation of happy facial expressions was compared with imitation of non-emotional facial expressions, we found significant activation peaks in the right hemisphere only. Concerning the insula, previous studies on imitation, execution or observation of emotional facial expressions report controversial results. Carr et al. [9] reported bilateral insula activation during observation but only left hemispheric activation during imitation of emotional facial expressions. This finding was underlined by van der Gaag and colleagues [13], who found bilateral insula activation when observation of emotional facial expressions was contrasted with observation of non-emotional facial expressions. Contrary, left hemispheric activation of the insula was found during observation and execution of pleasant facial affect by Hennenlotter and colleagues [14]. Lee et al. [10] noted significant left insula involvement during imitation of angry and licking, but right insula involvement during imitation of chewing facial expressions. Nevertheless, right insula activation in our study is in line with the finding that the magnitude of facial movement during imitation of pleasant affect predicts activation of the right insula [10] and with studies relating activation of the right insula to interoception [34], [35].

Previous imaging studies examining emotion processing reported increased amygdala activation more often in the left than in the right hemisphere (for a meta analysis see [54]; for a review see [55]). Contrary to this, we found increased activation of the right amygdala. Studies examining the putative human MNS often reported activation of the right amygdala during observation [9], [13], [14], execution [14] or imitation [9] of emotional facial expressions as well. Note that our previous analyses of shared representations of observation and execution of positive facial affect revealed increased activation of the right amygdala, too [20]. Moreover, right amygdala activation was assumed to be associated with rather automatic emotion processing (here mood induction), whereas increased left amygdala activation was found during cognitive, intentional emotion regulation [52].

Apart from these results, recent research suggests an influence of acquisition parameters of EPI sequences on laterality of amygdala activation. Mathiak and colleagues [56] investigated the influence of phase encoding direction on laterality of amygdala activation, assuming that lateralization in previous studies might have been due to intense medial temporal lobe susceptibility artefacts [56]. In this study, a significant interaction between hemisphere and phase encoding direction was noted when participants observed fearful faces masked with neutral faces. Increase of left amygdala activation was found for left to right but not for right to left side phase encoding. Authors stated that amygdala lateralization has to be discussed carefully until the EPI sequences are optimized. However, we found the same lateralization pattern also for the insula, which is not affected by susceptibility artefacts due to phase encoding direction. In total, lateralization of the activation of the amygdala and insula as part of the perception-execution matching system might be influenced by valence, task or cognitive load.

### Limitations

A limitation of this study is the use of a blocked design as we cannot exclude bias due to differential habituation of the amygdala response in different tasks. Furthermore, we compared a happy facial expression (a smile) to a non-emotional facial expression (lip protrusion). We tried to choose similar movements to compare happy with non-emotional facial expressions (in both expressions the lips are moved). Nevertheless, the amount of motion or differential usage of facial muscles to produce the two facial expressions may be possible confounds that are not excluded by our study design. Likewise, we cannot generalize the results to other emotions. Although both structures have been shown to be involved during observation (e.g. [13]) and imitation of different emotions [9], subsequent studies directly comparing the emotions in all three tasks are needed to clarify this issue. Particularly merest evidence concerns differences between the emotions during pure execution of emotional facial expressions as most studies investigated observation and/or imitation of facial expressions (e.g. [9], [19]).

### Conclusion

Our findings support the notion that insula and amygdala activation is increased in addition to activation in the putative human mirror neuron system during the imitation of positive facial expressions. We found increased activation of the right insula during imitation and observation of positive facial expressions, which may be related to enhanced emotional awareness during the presentation of emotionally salient stimuli. In contrast, the increase of right amygdala activation to happy in comparison to non-emotional facial expressions was larger during imitation and execution than to observation. This might be due to an enhanced autonomic reaction in response to feedback from facial muscles of the amygdala, or to differences in habituation of amygdala response. In addition, we found that the insula, but not the amygdala, showed enhanced activity during execution and imitation compared with observation of happy facial expressions.
